# Pemphigus Vulgaris and Bullous Pemphigoid: Update on Diagnosis and Treatment

**DOI:** 10.5826/dpc.1003a50

**Published:** 2020-06-29

**Authors:** Vito Di Lernia, Dahiana M. Casanova, Mohamad Goldust, Cinzia Ricci

**Affiliations:** 1 Dermatology Unit, Arcispedale Santa Maria Nuova, Azienda USL-IRCCS di Reggio Emilia, Italy; 2 University Guglielmo Marconi, Rome, Italy & Department of Dermatology, University Hospital, Basel, Switzerland

**Keywords:** pemphigus, pemphigoid, autoimmune, bullous, disorder

## Abstract

Autoimmune bullous disorders are a heterogeneous spectrum of skin disorders characterized by the production of autoantibodies against adhesion molecules of the skin. The 2 major groups of diseases are “pemphigus diseases” and “autoimmune bullous diseases of the pemphigoid type.” Pemphigus diseases are a group of autoimmune blistering diseases of the skin and mucous membranes characterized by intraepithelial cleft and acantholysis. The main subtypes of pemphigus include pemphigus vulgaris, pemphigus foliaceus, and paraneoplastic pemphigus. Diagnosis is based on clinical manifestations and confirmed with histological, immunofluorescence, and serological testing. Recently multivariant enzyme-linked immunosorbent assay systems have been developed as practical screening tools for patients with suspected autoimmune bullous dermatoses. The current first-line treatment of pemphigus is based on systemic corticosteroids that are often combined with immunosuppressive adjuvants, such as azathioprine, mycophenolate mofetil, and the anti-CD20 monoclonal antibody rituximab, usually at initiation of treatment. Rituximab efficacy is higher when it is administered early in the course of the disease. Therefore, it should be used as first-line treatment to improve efficacy and reduce cumulative doses of corticosteroids and their side effects. Treatment of bullous pemphigoid is based on disease extension. Localized and mild forms can be treated with superpotent topical corticosteroids or with nonimmunosuppressive agents. In patients with generalized disease or whose disease is resistant to the treatments described above, systemic corticosteroids are preferred and effective. Adjuvant immunosuppressants are often combined with steroids for their steroid-sparing effect.

## Introduction

Autoimmune bullous disorders (ABDs) encompass a number of heterogeneous conditions linked by the loss of tolerance to structural proteins of the skin. As a consequence of breakdown of tolerance, autoantibodies targeting epidermal or subepidermal adhesion proteins are produced. The loss of adhesion between keratinocytes or between basal keratinocytes and the underlying epidermal basement membrane leads to an impaired resilience of the epidermis resulting in intraepithelial or subepithelial blisters and erosions of the skin and mucous membranes. ABDs are a major cause of severe morbidity and considerable mortality [1].

Based on the available literature data, this paper aims to provide an up-to-date overview on diagnosis and therapy of pemphigus vulgaris (PV) and bullous pemphigoid (BP), which represent the 2 major diseases in the heterogeneous clinical spectrum of ABDs.

## Classification

The classification of an ABD relies on the level of blistering and considers 2 major groups of diseases, namely “pemphigus diseases”(PDs) and “ABDs of the pemphigoid type.”

PDs are characterized by the production of pathogenic autoantibodies directed against different proteins of the desmosome, leading histologically to intraepithelial blistering. There are several variants of pemphigus, but the 3 major forms include PV, pemphigus foliaceus (PF), and paraneoplastic pemphigus (Table 1). Pemphigus is driven by pathogenic antibodies to both desmoglein (Dsg) 1 and 3 (PV, mucocutaneous type), or Dsg3 (PV, mucosal dominant-type), or Dsg1 (PF). Numerous antigens are involved in paraneoplastic pemphigus (Table 2).

ABDs of the pemphigoid type or autoimmune subepidermal blistering diseases of the skin and mucosae constitute a large group of diseases characterized by the production of circulating autoantibodies against several structural proteins of the basement membrane zone, leading histologically to subepidermal blistering. The main disorders include BP, pemphigoid gestationis, mucous membrane pemphigoid, epidermolysis bullosa acquisita, and anti-p200 pemphigoid [2] (Table 3). BP is characterized by the generation of autoantibodies directed in particular against BP180/collagen XVII and BP230/dystonin.

## Epidemiology

Prospective studies suggest the incidence rates of ABDs are in the range of 14.5–20.4/million [3–5]. Most of the available epidemiological data derive from PV, the most frequently reported disorder among the PDs, and BP [3].

PV incidence appears to be highly variable according to geographic regions and ethnic groups. The incidence rates reported in European prospective studies range between 0.5 and 4.0/million [5,6]. Higher rates, up to 16.1/million/year, have been observed in subjects from Israel and Iran [7,8]. In fact, the disease is more common among individuals of Ashkenazi origin, but also in ethnic groups from Iraq and Iran. Therefore, ethnic differences should be taken into account when comparing incidence rates in countries with populations of Jewish heritage [3,9]. Pemphigus is most frequently diagnosed between ages 50 and 60 in European countries, but it can be seen earlier outside of Europe.

The yearly incidence of BP reported in European prospective studies varies between 2.5 and 13/million [5,6]. The largest series of patients (N = 869) collected in a retrospective historical cohort from the UK showed higher incidence rates of 42.8/million [10]. Rising incidence rates of BP in Europe have been linked to risk factors, including population aging; drugs, in particular antidiabetics; diuretics; phenothiazines; major cognitive impairment; and disabling neurological disorders [3,11]. BP affects mostly the elderly: it is generally seen in individuals older than 70 years. Overall incidence is higher in females [3].

## Genetics

There is evidence that genetics plays a critical role in PV development, severity, and prognosis. An increased frequency of HLA-DRB1*04:02 haplotypes in Ashkenazi Jewish patients [12] and DQB1*05:03 in non-Jewish Caucasian populations [13] has been observed. The coexistence of alleles of DRB1*14/DQB1*05 and A*11/DQB1*05 would be strongly influential for the predisposition to the disease, while that of HLA-B*50/DQB1*02 could play a protective role [14]. In addition, multiple single nucleotide polymorphisms within different genes may confer susceptibility to the diseases, probably in a population-specific way [15].

Serological typing for HLA class II antigens in patients with ABD of the pemphigoid type revealed a highly significant association with HLA-DQβ1*0301 [16].

## Pathophysiology

PV and BP are characterized by the loss of tolerance to autoantigens expressed primarily in the skin, in particular desmosomal proteins in PV and components of the hemidesmosomes in BP.

In PDs, the production of pathogenic immunoglobulin G (IgG) autoantibodies, mainly IgG4, against the desmosomal cadherins Dsg1 and Dsg3, causes loss of epidermal keratinocyte adhesion. Dsg3-specific autoreactive T cells are deleted in periphery, and regulatory T cells (Tregs) play an important role in such peripheral tolerance [17]. Environmental factors, in particular pathogens, have been hypothesized as causes of reverting immunological tolerance with resulting autoantibody production [18]. A possible link with rotavirus infection has been suggested because of cross-reactive VH1-46 antibodies, which are able both to disrupt keratinocyte adhesion and inhibit rotavirus replication [19]. An additional relationship between the exposure to a noninfectious environmental antigen and the development of an autoimmune response to self-antigen has been documented in fogo selvagem, the endemic form of PF. In this condition, cross-reactive epitopes on Dsg1 and LJM11 sand fly salivary gland antigen could drive the production of pathogenic IgG4 autoantibodies anti-Dsg1 [20].

Although antibody-mediated disease mechanisms in PV are widely characterized, the T cell implication has to be better clarified. Autoreactive CD4+ T lymphocytes have been implicated in the regulation of the production of pathogenic anti-Dsg3 autoantibodies by B cells [21]. There is a strong association between PV and distinct major histocompatibility complex class II haplotypes, which are considered essential for the presentation of specific Dsg3 peptides to autoreactive CD4+ helper T cells [18,22].

The causes of loss of tolerance leading to the production of antibodies anti-BP180/collagen XVII and BP230/dystonin, and less frequently also against other antigens in BP, are not known. BP has been linked to environmental factors as physical agents, such as radiation therapy, ultraviolet radiation [23] trauma, neurodegeneration or neuroinflammation [24] and drugs, in particular gliptins [25]. Muramatsu et al demonstrated that Tregs are essential in preventing the spontaneous production of BP autoantibodies as well [26]. An imbalance between autoreactive helper T cells and Tregs and a T cell-independent activation of a toll-like receptor system could underlie a B cell stimulation, with resulting BP autoantibody production [24].

## Clinical Presentation

**Figure 1:**
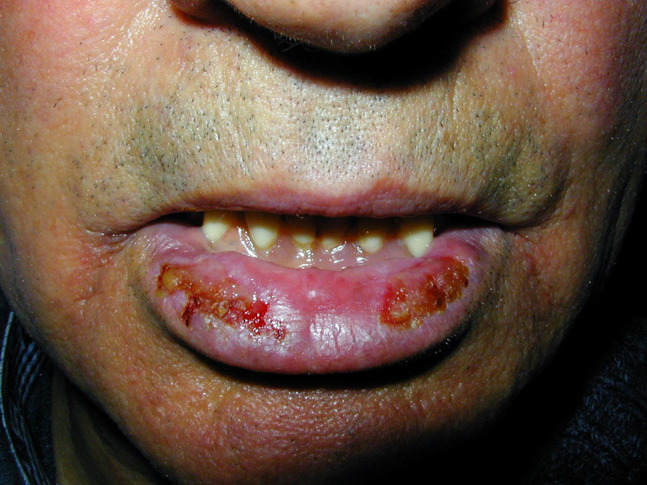
Solitary lip ulcerations as highly unusual manifestations of pemphigus.

**Figure 2:**
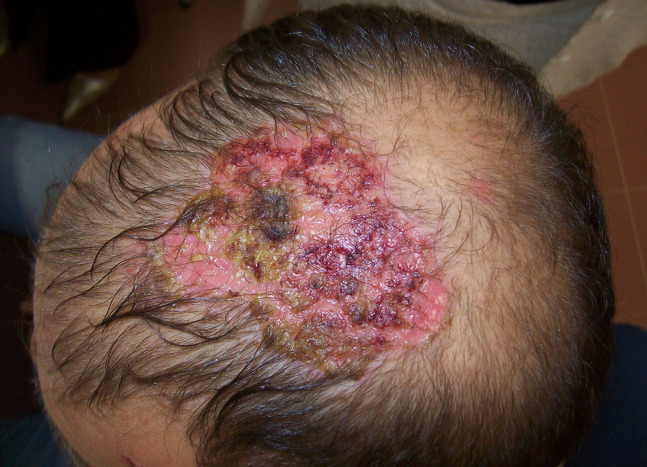
Erosions, crusted and scaly plaques in a case of pemphigus of the scalp. The scalp shows an abundance of desmogleins in hair follicles and may be the first location of the disease.

PV, the most frequent and severe form of pemphigus, affects both skin and mucosal surfaces. The disease often begins in the oral mucosa with superficial, flaccid blisters that rapidly rupture, leaving slow-healing, painful, moist erosions that may make oral intake difficult and cause pain and fetor as well. Accompanying symptoms are sialorrhea and bloody saliva. Involvement of other skin areas may occur from weeks to months later. In addition to the cutaneous and mucosal surfaces of the mouth (Figure 1), the pharynx, vocal folds, and anogenital mucosa may also be affected. Scalp involvement is observed in up to 60% of patients (Figure 2). Clinical variants include mucosal-only disease (which correlates with circulating anti-Dsg3 autoantibodies), cutaneous-only disease (anti-Dsg1 antibody predominant), or more frequently, mucocutaneous involvement (presence of both anti-Dsg1 and anti-Dsg3 antibodies). Recently, clinical scores of disease severity have been implemented. The Pemphigus Disease Area Index and Autoimmune Bullous Skin Disorder Intensity Score have been recognized as robust tools to correctly assess disease activity [27].

**Figure 3:**
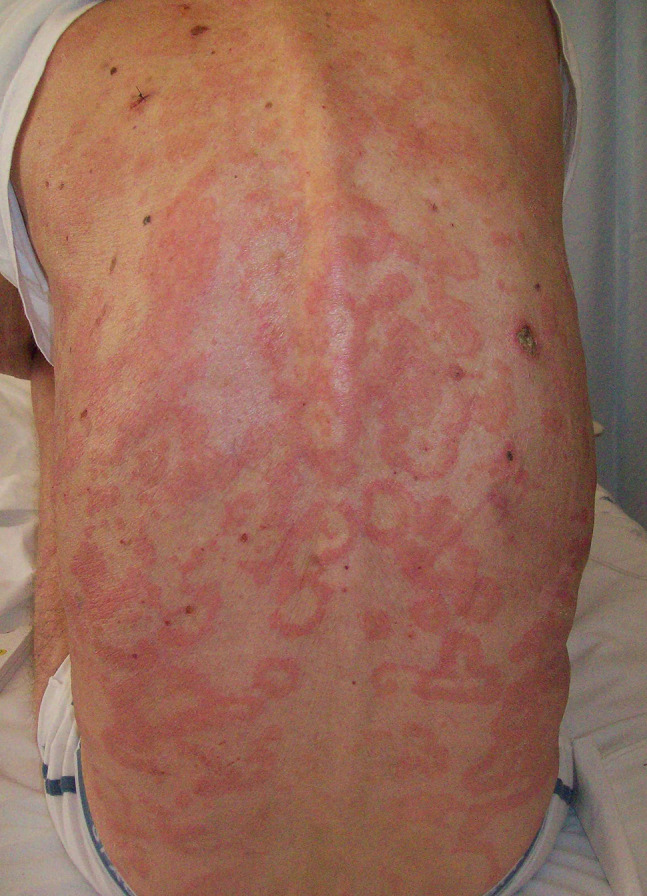
Numerous polycyclic and targetoid lesions during the nonbullous phase of bullous pemphigoid.

**Figure 4:**
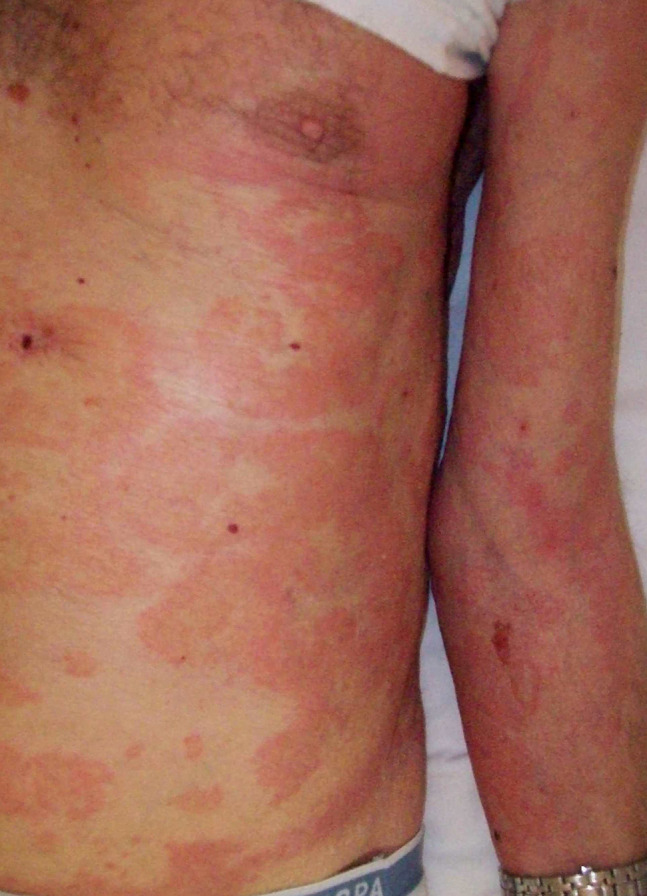
Urticarial and polycyclic lesions of the nonbullous phase of bullous pemphigoid associated with a tense bulla on the flexor surface of a forearm.

**Figure 5:**
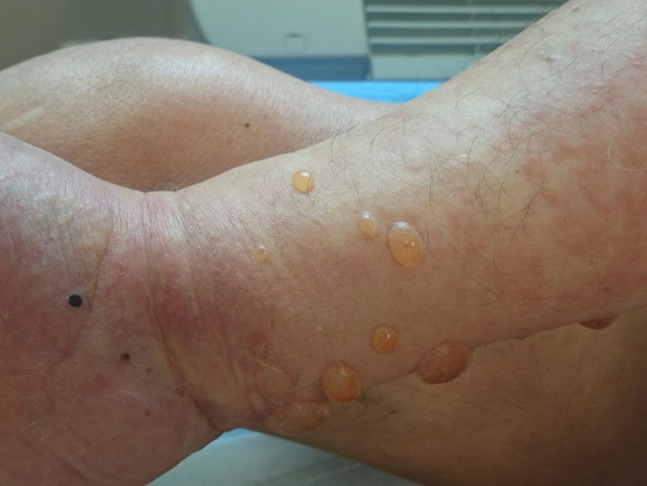
Typical tense bullae on an erythematous base in a patient with bullous pemphigoid.

Classic BP affects elderly individuals, usually above age 70 years. The spectrum of clinical presentation is highly variable. In a consistent proportion of patients, the blistering eruption is preceded by a prodromal nonbullous phase, usually lasting weeks to months and even, in rare cases, remaining the only manifestation of the disease [28]. During the nonbullous phase, pruritic, erythematous, or urticarial patches and plaques occur. Also eczematous, polycyclic, targetoid, nodular, or lichenoid lesions may be observed (Figure 3). The bullous phase is characterized by tense bullae on an erythematous, urticarial base, localized or widespread (Figures 4 and 5). Pruritus is common and often severe. After rupture, bullae leave moist erosions and crusts that resolve without scarring. Mucosal involvement may be observed in 10% to 30% of the cases with oral, esophageal, and genital involvement. Two disease scores, the BP Disease Area Index and the Autoimmune Bullous Skin Disorder Intensity Score, have shown significant reliability, validity, and responsiveness [29-31]. BP shows a different clinical course in infants and children. Acral involvement is common, especially on the face (62%), palms and soles (79%); localized lesions on the genital area may be observed in 17% of cases [32].

## Diagnosis

Clinical presentations of ABDs often overlap, and diagnosis may not be easily made on the basis of clinical features alone. Therefore, ABDs are usually diagnosed using 3 criteria: (1) the overall clinical picture, including patient history and physical examination; (2) histopathology; and (3) a positive direct immunofluorescence (DIF) microscopy, usually performed on perilesional skin, or serological detection of autoantibodies against the involved epithelial antigens [22]. Immunodiagnostic tests are particularly useful to differentiate the various diseases.

### Histopathology

#### Pemphigus Vulgaris

Biopsy should be taken in an early intact blister. When only erosions are present, as usually happens in the oral area, a biopsy should be obtained from the active border of a denuded area. The characteristic histological finding is an intraepidermal blister caused by acantholysis, which consists of the separation of keratinocytes just above the basal cell layer due to a loss of the normal cell attachment. In the cavity of the blister, a few inflammatory cells, in particular eosinophils, may be observed. The surrounding inflammation is minimal [22]. Eosinophilic spongiosis may be the only histological feature of pemphigus in the initial stages. In such cases eosinophils invade a spongiotic epidermis without evidence of acantholysis.

Single or grouped acantholytic cells can be quickly documented by cytological examination (Tzanck cytodiagnostic test). After opening of an intact roof of a blister, material has to be scraped from the base of a vesicle or blister and smeared onto a microscopic slide. After air-drying, samples are commonly stained with May-Grünwald-Giemsa and evaluated. This test does not replace histological examination, since acantholytic keratinocytes may also be observed in other dermatoses as a secondary phenomenon to inflammation or ballooning degeneration.

#### Bullous Pemphigoid

The histopathological assessment of an early bulla shows a subepidermal blister containing a net of fibrin with a variable number of eosinophils and/or neutrophils accompanied by a dermal inflammatory infiltrate mainly consisting of eosinophils and neutrophils [3,33]. In the nonbullous phase, histopathological findings may be nonspecific, since only subepidermal clefts and eosinophilic spongiosis may be observed [34].

### Direct Immunofluorescence

DIF is the most reliable and sensitive diagnostic test for ABDs for the most part. However, nonspecific staining may occasionally be seen in other cutaneous disorders with occasional false-positive findings [35,36]. DIF investigates the skin or mucous membrane and shows antibody deposition on the keratinocyte cell surface (in PDs) or along the basement cell membrane (in BP).

#### Pemphigus Vulgaris

Biopsy of perilesional skin or mucosa shows deposits of IgG at the keratinocyte cell membrane [22,37]. IgG deposition is seen in up to 100% of patients with active disease. Complement (C3) deposition may not be observed, since IgG4, the dominant subclass of IgG involved in PV, does not fix complement.

#### Bullous Pemphigoid

It is best to biopsy perilesional skin from a recent blister. The diagnostic hallmark consists of fine, linear, continuous deposits of IgG and/or C3 along the dermal-epidermal junction. Occasionally IgA and IgE may be observed showing a similar pattern [33,38]. Salt-split technique is a useful tool in the differential diagnosis of other ABDs, such as epidermolysis bullosa acquisita, which shows a similar DIF pattern. In BP, immune deposits are found in the epidermal side or mixed at both the epidermal and dermal sites of the split (n-serration pattern), while in epidermolysis bullosa acquisita, deposits are typically localized on the dermal side of the cleavage (u-serration pattern).

### Serological Studies

The method of serological detection of the autoantibodies largely relies on indirect immunofluorescence (IIF) and enzyme-linked immunosorbent assay (ELISA) tests. Recently, novel diagnostic multivariant assays were developed as practical screening tools for patients with suspected ABD with the aim of processing the most common autoantibodies simultaneously [39,40].

#### Pemphigus Vulgaris

IIF allows for the detection of circulating autoantibodies against proteins or epithelial keratinocytes by incubating patient serum with appropriate commercially available substrates containing the target antigen. The testing study is conducted on monkey esophagus or epithelial substrates. PV sera produces a characteristic smooth and reticular pattern on most epithelial layer, referred as “fishnet-like,” “chicken wire,” or “honeycomb” pattern [37,38].

Cloning of the gene coding for the major pemphigus antigens, Dsg1 and Dsg3, has enabled the production of recombinant proteins, which are used to detect IgG autoantibodies by ELISA [41]. The Dsg3/Dsg1 autoantibody profile defines the clinical outcome, since PV with exclusive involvement of the mucous membranes is associated with IgG against Dsg3, while the mucocutaneous variant of PV is associated with both anti-Dsg1 and anti-Dsg3 IgG. The detection of IgG autoantibodies by ELISA is positive in more than 90% of cases [37]. The titers of serum IgG autoantibodies against Dsg1 and Dsg3 generally correlate with the extent and clinical activity of disease, and high levels of anti-Dsg1 by ELISA has a positive predictive value for skin relapses [37]. Therefore, ELISA may represent a good serological marker of disease activity, although evidence about its predictive value from large prospective cohort studies is lacking [37]. One must keep in mind that anti-Dsg antibodies have been occasionally discovered in sera of normal patients and those affected with other bullous diseases [41]. In addition, a small number of pemphigus patients may not show the PV phenotype expected by their Dsg autoantibody serum profile [42].

#### Bullous Pemphigoid

IIF may demonstrate circulating IgG antibodies binding to the basal membrane. The most specific test substrate for BP is salt-split skin, which is healthy human skin in which subepidermal splitting was induced by 1 mol/L sodium chloride solution [43]. Other substrates include monkey or rabbit esophagus, with possible lower sensitivity [38].

ELISA test may show anti-BP180 and anti-BP230 IgG antibodies. The serum level of anti-BP180 antibodies may be monitored during the course of the disease. High titers of anti-BP180 NC16A IgG after therapy cessation are considered a predictor of risk of relapse. Even then, the test result may be positive in healthy individuals or in individuals with other pruritic, inflammatory skin disorders [38].

## Therapeutic Management

Because of their chronic nature, ABDs may last for a lifetime or for several years with a high tendency to relapse. PV is a potentially life-threatening skin disorder that requires early recognition and prompt treatment. BP is highly associated with old age, distinct drugs, and neurological and psychiatric diseases. Treatment depends on the extent and severity of disease. In both conditions the primary objective is to promote healing of the bullous and erosive cutaneous and/or mucous lesions. Additional goals are to reduce itch, prevent or reduce the recurrences of the blistering eruptions, improve the quality of life of patients, and minimize and identify as quickly as possible serious side effects associated with long-term treatment, particularly in the elderly [37,38]. A complete medical history focusing on drug history and comorbid diseases that may affect treatment decisions is mandatory. A complete biochemistry screening should be conducted and should include liver and renal function tests.

## Treatment

### Pemphigus Vulgaris

The morbidity and mortality of PV have remained high owing to complications of therapy, such as pneumonia, septicemia, and cardiovascular disease. Therefore, correct management of the comorbidities of pemphigus is crucial to decrease disease-associated morbidity and mortality. The traditional treatment paradigm of PV relies on systemic corticosteroid immunosuppression. Prednisone is usually given at 0.5–1.5 mg/kg/day as a typical initial dosage. The therapeutic effect is evaluated by the number of new blisters per day and the ability to stop the blistering eruption. In refractory cases, the initial dose may be increased to 2 mg/kg/day. On the other hand, if clinical remission is obtained and no new blisters occur, glucocorticoids may be gradually tapered. Appropriate steroid-tapering strategies may prevent relapses, although an optimal treatment regimen remains indeterminable because of the lack of evidence. A decrease in the titers of circulating autoantibodies may drive tapering decisions. Recommendations by an international expert panel are to check serum autoantibodies at the initiation of treatment, after 3 months and every 3–6 months, or in case of relapse [44]. Control of disease activity is usually achieved within several weeks, whereas complete remission of quiescent disease on minimal treatment (≤10 mg/day prednisone) more often requires several months. Long-term, complete remission off treatment may require years of therapy [45].

Additional immunosuppressive adjuvants, such as azathioprine (1–3 mg/kg/day) or mycophenolate mofetil (2 g/day), are often administered at initiation of treatment or in refractory cases in order to achieve faster or better control of the disease and spare corticosteroids, respectively. Second-line adjuvants include cyclophosphamide, methotrexate, intravenous immunoglobulins, and immunoadsorption. The latter treatment consists of the passive removal of IgG from the patient’s systemic circulation. When complete remission is obtained with combined therapy, the dosage of the immunosuppressive adjuvant drug is continued, while the prednisone is gradually tapered. No particular advantage has been found with intravenous corticosteroid pulse treatment over treatment with oral corticosteroids [46].

Rituximab, a chimeric monoclonal antibody targeting the CD20 antigen of B lymphocytes, has recently emerged as a highly promising therapeutic option for PV. Rituximab causes B-cell depletion and a subsequent reduction in pathogenic autoantibodies. Pretreatment investigations, contraindications, and treatment schedules are summarized in Table 4. Rituximab is one of the only drugs to gain European Medicine Agency and US Food and Drug Administration approval for the treatment of PV. Previous clinical experiences showed its dramatic efficacy as second- or third-line treatment in severe recalcitrant or relapsing cases of PV [47,48]. In particular, a meta-analysis including 578 patients with pemphigus showed a remission rate of 76% following a single cycle of rituximab, with a relapse rate of 20% (2 years) to 60% (5 years) [49]. More robust evidence showed a higher efficacy when it was administered early in the course of the disease [50]. Ritux 3, a prospective, open-label, randomized clinical trial investigated rituximab as first-line treatment in combination with short-term prednisone vs prednisone alone for the treatment of pemphigus. Results showed that first-line use of rituximab plus short-term prednisone for patients with pemphigus is more effective than using prednisone alone, with fewer adverse events [50]. At month 24, 89% of 46 patients assigned to rituximab plus short-term prednisone were in complete remission off therapy vs 34% of 44 assigned to prednisone alone. A post hoc analysis of the enrolled patients in the Ritux 3 study showed a steroid-sparing effect of rituximab, with rituximab-treated patients having lower cumulative doses of corticosteroids and experiencing less severe or life-threatening corticosteroid-related adverse events [51]. Therefore, intravenous rituximab is now recommended as first-line option for new-onset moderate to severe pemphigus, but also for previously treated patients who do not achieve clinical remission with systemic corticosteroids and/or immunosuppressive adjuvants [44,52].

**Figure 6:**
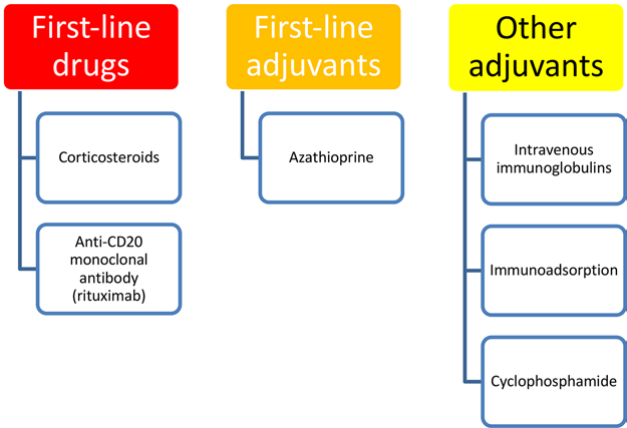
Treatment options for pemphigus.

The introduction of rituximab presents an opportunity to change the traditional therapeutic approach to PV. A complete remission off therapy may represent a realistic expectation for many patients. Many aspects remain to be deepened. B-cell repopulation, low CD41 T-cell count, persistence of anti-Dsg1 (>20 IU) and Dsg3 (>120 IU) at month 3, and severe Pemphigus Disease Area Index score at baseline (>45) were identified as predictors of relapse in patients treated with rituximab [53,54]. Additional areas that deserve further studies are the combination protocols with corticosteroids, the possibility of using CD20 inhibitors alone, prevention of relapses, the role of anti-Dsg ELISA values as biomarkers to drive further infusions, and the benefit from combining other adjuvants in patient management. Consequently, the therapeutic algorithm of PV could be redefined in the future (Figure 6).

Topical therapy of PV is essentially symptomatic and is practiced to alleviate inflammation and prevent secondary infections. Usually corticosteroids, calcineurin inhibitors or corticosteroids and antibiotics in combination are administered on the cutaneous and mucous lesions. Cutaneous erosive lesions should be covered using low-adhesive wound dressings. Supportive care for oral lesions includes gel-containing local anesthetics and proper dental care.

Limited experience proved rituximab useful in refractory PF as well [55,56].

### Bullous Pemphigoid

**Figure 7:**
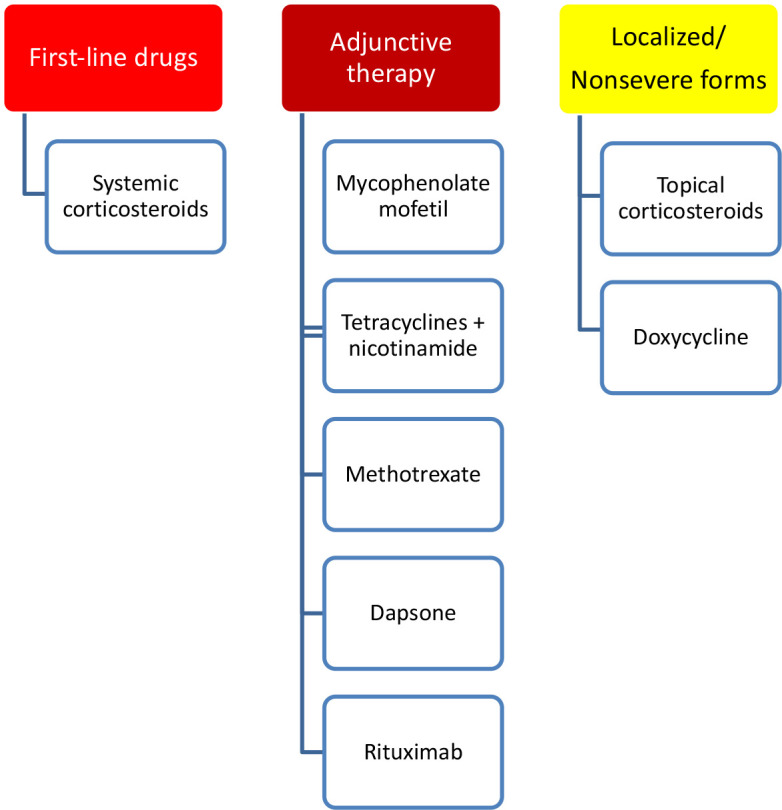
Treatment options for bullous pemphigoid.

Although there is no generally accepted classification of disease severity, a classification of BP into mild (<10% affected body surface area), moderate, and severe forms has been suggested [57]. The effectiveness of topical therapy with superpotent topical corticosteroids in localized and moderate forms of BP is supported by a Cochrane review [38,58]. Be aware that the dose of 40 g every day or 10 to 30 g every day [59,60], which has proven equally effective as systemic prednisolone, is equivalent to about a tube per day. Practicability and limitations of this drug regimen are that for some patients with BP, a twice-daily topical application [57] on widespread areas or the daily purchase of an ointment tube may not be manageable. In the localized, nonsevere forms, immunomodulatory, nonimmunosuppressant drugs such as doxycycline may be considered a viable option [61]. Although its exact mechanism of action is not well understood, doxycycline proved to be not inferior to oral prednisolone for short-term blister control and is safer in the long term [61,62]. Systemic corticosteroid therapy (prednisone 0.5 mg/kg/day) represents the treatment of choice for severe forms [38]. For maintenance treatment, doses may be tapered gradually within 4 to 6 months of initiation of treatment. Adjunctive therapy includes combination with azathioprine, mycophenolate mofetil, tetracyclines plus nicotinamide, methotrexate, and dapsone [63] (Figure 7). Bacterial superinfection of erosions should be treated with local antiseptics. Wound dressings should be considered for large wounds. Sterile puncture of large blisters is recommended [57]. Available data suggest that rituximab may provide clinical benefits for patients with refractory BP [64], although with less effectiveness. A complex interplay of complement activation of IgG autoantibodies deposited at the dermal-epidermal basement membrane zone and inflammatory response might be not fully ruled by a B-cell downregulation and a decrease in autoantibody titers [65].

Recent studies support a pathogenic role of IgE in the development of BP. This hypothesis is endorsed by the finding of IgE deposition in the basement membrane zone in patients with BP. In addition, there is correlation between serum levels of IgE autoantibodies against BP180 and BP disease activity [62]. Use of IgE targeted therapies, such as omalizumab, has been shown as promising in recent studies [66], with 80% complete response rates and mean time to recurrence of 3.4 months [64].

## Conclusions

Current treatment paradigms in ABD are traditionally based on the administration of immunosuppressive drugs, often in combination. The advent of rituximab, the monoclonal IgG antibody against CD20, has revolutionized the treatment of pemphigus, indicating that a complete remission off therapy is now possible. Therefore, additional studies are investigating other anti-CD20 monoclonal antibodies [67]. Ideally, therapy for autoimmune diseases should eliminate pathogenic autoimmune cells without affecting protective immunity. Innovative approaches include antigen-specific immune suppression in PV by therapeutic immunoadsorption of pathogenic autoantibodies [68,69]. A promising approach is the use of reengineering chimeric autoantibody receptor T cells. Patient-derived T cells are modified ex vivo to express a chimeric antibody receptor, which allows selective recognition and consequent killing of anti-Dsg3 autoreactive B lymphocytes [70,71]. Also in BP, new therapeutic targets aim to provide new treatment strategies that may go beyond nonspecific immunosuppression. In addition to omalizumab, monoclonal antibodies to IL-5 such as mepolizumab and bertilimumab, an anti-eotaxin-1 antibody, are currently being investigated in BP [65]. In the context of immunomodulatory drugs, dimethyl fumarate, which is a prodrug utilized in psoriasis and multiple sclerosis, is also under investigation for BP because of its pleotropic anti-inflammatory effects [72].

## Figures and Tables

**Table 1:** Classification of Pemphigus

Type	Variants
Pemphigus vulgaris	Pemphigus vegetans Pemphigus herpetiformis
Pemphigus foliaceus	Fogo selvagem (pemphigus brasiliensis) Pemphigus erythematosus
Paraneoplastic pemphigus	
Atypical pemphigus	
“Drug-induced” pemphigus	
IgA pemphigus	

Ig = immunoglobulin.

**Table 2:** Classification and Autoantigens in Pemphigus Group Diseases

Diseases	Ig	Antigen

Pemphigus vulgaris, mucosal dominant type	IgG	Dsg3
Pemphigus vulgaris, mucocutaneous type	IgG	Dsg3 + Dsg1

Pemphigus vegetans	IgG	Dsg3, Dsg1, Dsc3

Pemphigus herpetiformis	IgG	Dsg1, (Dsg3), Dscs

Pemphigus foliaceus	IgG	Dsg1

Pemphigus erythematosus	IgG	Dsg1

Paraneoplastic pemphigus	IgG	Plectin, epiplakin, desmoplakin I/II, BP230, envoplakin, periplakin, Dsg3, Dsg1, Dscs, α-2-macroglobulin-like-1

IgA pemphigus, subcorneal pustular dermatosis type	IgA	Dsc1
IgA pemphigus, intraepidermal neutrophilic type IgA dermatosis	IgA	Unknown

BP = bullous pemphigoid; Dsc = desmocollin; Dsg = desmoglein; Ig = immunoglobulin.

**Table 3:** Classification and Autoantigens in Autoimmune Bullous Disorders of the Pemphigoid Type

Diseases	Ig	Antigen
Bullous pemphigoid	Ig	BP180, BP230
Pemphigoid gestationis	Ig	BP180, BP230
Linear IgA dermatosis	IgA	BP180
Mucous membrane pemphigoid	IgG/IgA	BP180, BP230, laminin 332, α6β4 integrin
Anti-laminin γ-1 pemphigoid	IgG	Laminin γ-1(p200)
Lichen planus pemphigoid	IgG	BP180, BP230
Epidermolysis bullosa acquisita	IgG	Type VII collagen
Dermatitis herpetiformis	IgA (IgG)	Epidermal transglutaminase, tissue transglutaminase, deamidated gliadin

BP = bullous pemphigoid; Ig = immunoglobulin.

**Table 4:** Dosage and Pretreatment Investigations for Rituximab in Pemphigus Vulgaris

Rituximab Dosage	Pretreatment Investigations	Contraindications	Adverse Events
Two 1,000-mg intravenous infusions separated by 2 weeks [1]; lower doses (500 mg) may be used for retreatment (at month 12 and every 6 months thereafter); methylprednisolone 100 mg intravenous or equivalent glucocorticoid is recommended 30 minutes prior to each infusion	Complete blood cell count, liver and renal function tests, creatinine, interferon gamma release assay, hepatitis B and hepatitis C markers, HIV antibodies, chest X-ray, ECG, cardiological examination	Active, severe infections, severely immunocompromised state, severe heart failure (New York Heart Association class IV) or severe, uncontrolled cardiac disease, cardiomyopathy, ischemic heart disease, severe arrhythmias such as rapid atrial fibrillation, frequent premature ventricular contractions	Infusion reactions, depression, infections, cardiac disorders, rare cases of fatal progressive multifocal leukoencephalopathy

The dosing protocol corresponds to that of the European Medicine Agency’s “Summary of product characteristics” and US Food and Drug Administration’s highlights of prescribing information. Different dosing protocols are reported in expert recommendations and therapeutic guidelines [42,50].
